# Combination of Pirfenidone and Andrographolide Ameliorates Hepatic Stellate Cell Activation and Liver Fibrosis by Mediating TGF-*β*/Smad Signaling Pathway

**DOI:** 10.1155/2024/2751280

**Published:** 2024-06-21

**Authors:** Guang Xu, Tidong Ma, Chonggao Zhou, Fan Zhao, Kun Peng, Bixiang Li

**Affiliations:** Department of Neonatal Surgery Hunan Children's Hospital, Changsha 410007, China

## Abstract

**Background:**

Biliary atresia (BA) is a devastating congenital disease characterized by inflammation and progressive liver fibrosis. Activation of hepatic stellate cells (HSCs) plays a central role in the pathogenesis of hepatic fibrosis. Our study aimed to investigate the pharmacological effect and potential mechanism of pirfenidone (PFD) and andrographolide (AGP) separately and together on liver fibrosis of BA.

**Materials and Methods:**

The bile ducts of male C57BL/6J mice were ligated or had the sham operation. The *in vivo* effects of PFD and/or AGP on liver fibrosis of BA were evaluated. Human hepatic stellate cells (LX-2) were also treated with PFD and/or AGP *in vitro*.

**Results:**

PFD and/or AGP ameliorates liver fibrosis and inflammation in the mice model of BA, as evidenced by significant downregulated in the accumulation of collagen fibers, hepatic fibrosis markers (*α*-SMA, collagen I, and collagen IV), and inflammatory markers (IL-1*β*, IL-6, and TNF-*α*). Moreover, compared with monotherapy, these changes are more obvious in the combined treatment of PFD and AGP. Consistent with animal experiments, hepatic fibrosis markers (*α*-SMA, collagen I, and CTGF) and inflammatory markers (IL-1*β*, IL-6, and TNF-*α*) were significantly decreased in activated LX-2 cells after PFD and/or AGP treatment. In addition, PFD and/or AGP inhibited the activation of HSCs by blocking the TGF-*β*/Smad signaling pathway, and the combined treatment of PFD and AGP synergistically inhibited the phosphorylation of Smad2 and Smad3.

**Conclusion:**

The combined application of PFD and AGP exerted superior inhibitive effects on HSC activation and liver fibrosis by mediating the TGF-*β*/Smad signaling pathway as compared to monotherapy. Therefore, the combination of PFD and AGP may be a promising treatment strategy for liver fibrosis in BA.

## 1. Introduction

Biliary atresia (BA) is a potentially devastating congenital disease, leading to progressive occlusion of the extrahepatic and the intrahepatic biliary tract, causing progressive inflammation and liver fibrosis, and eventually leading to death in the first year of life [1]. In children and adults, the progress of liver fibrosis in BA is faster than in any other liver or biliary disease [2]. Although the Kasai procedure has greatly improved prognosis, most patients need liver transplantation, accounting for about half of the transplants in children [3]. The potential pathogenic factors of the disease have not yet been fully elucidated. Considering the complex etiologies behind BA, liver fibrosis is far more than biliary cirrhosis secondary to biliary obstruction.

Fibrosis is no longer regarded as static but the process of continuous remodeling. Liver fibrosis is a reaction to chronic liver injury, which is characterized by excessive production and deposition of extracellular matrix (ECM). Hepatic stellate cells (HSCs) are the main cells that produce ECM, which transform into myofibroblast-like cells, thus producing a great number of ECM proteins such as *α*-SMA and collagen I, leading to hepatic fibrosis and subsequent cirrhosis [4]. Connective tissue growth factor (CTGF) is a member of the CTGF/CYR61/NOV (CCN) family and plays an important role in the formation of liver fibrosis [5]. TGF-*β*1 is the most widely studied subtype in liver fibrosis, and it is considered an effective cytokine for promoting fibrosis [6]. TGF-*β*/Smad signaling pathway plays an important role in the occurrence and development of liver fibrosis, and inhibition of the TGF-*β*/Smad pathway can alleviate liver fibrosis [7].

Inflammatory reactions aggravate the damage to the liver and bile duct and promote the formation of inflammatory mediators. The formation of inflammatory mediators, cytokines, and necrotic substances plays an important role in promoting the development of liver fibrosis [2]. The study of liver cytokines in BA found that the expression of IL-1*β*, IL-6, TNF-*α*, and TGF-*β*1 increased in peripheral blood of BA children. These factors can promote the increase of Th17 cells and other effector cells, suggesting that there is a proinflammatory microenvironment in the liver parenchyma of BA children [8, 9]. Various cytokines can stimulate the activation of HSCs, participate in the development of chronic inflammation, and then promote the formation of bile fibrosis, liver fibrosis, and cirrhosis [10].

So far, antihepatic fibrosis therapy represents an unsolved field of drug development, with great potential. Conventional or synthetic drugs for liver fibrosis disease management have serious adverse effects, especially when given for a long time. Therefore, the combined use of natural drugs in improving inflammation and fibrosis has been proposed as a mainstream choice to fight liver fibrosis. Pirfenidone (PFD) is one of the compounds recently approved for clinical use, and its main indication is idiopathic pulmonary fibrosis. Pirfenidone inhibited the differentiation of human lung fibroblasts into myofibroblasts induced by TGF-*β*1 *in vitro*, which showed that it inhibited the proliferation of TGF-*β*1 in a dose-dependent manner [11]. Andrographolide (AGP) is a natural diterpenoid compound, which is extracted from the aerial parts of *Andrographis* plants [12]. Andrographolide has many biological effects, including anti-inflammatory, antioxidation, and antitumor characteristics [13]. It has been reported that AGP ameliorates liver fibrosis in carbon tetrachloride-induced fibrotic mice [14]. However, there is no report on whether the combined treatment of PFD and AGP has a better antihepatic fibrosis effect. Therefore, this study aimed to explore the ameliorative effects of the combination of PFD and AGP in the regression of HSC activation induced by TGF-*β*1 and liver fibrosis. In addition, we studied the potential mechanisms of this process and evaluated the participation of the TGF-*β*/Smad pathway.

## 2. Materials and Methods

### 2.1. Cell Lines and Cell Culture

Human hepatic stellate cells (LX-2) applied in this experiment were provided by the cell bank of the Chinese Academy of Science (Shanghai, China). LX-2 cells were grown in an incubator in Dulbecco's Modified Eagle's medium (DMEM) (Thermo Fisher, MA, USA) complemented with 10% fetal bovine serum (FBS) (Sangon Biotech, Inc., Shanghai, China), penicillin (100 U/mL), streptomycin (100 U/mL), and recombinant human insulin (0.1 U/mL) at 37°C in 5% CO_2_. Cells were divided into five groups: the control group, TGF-*β*1 group, PFD group, AGP group, and PFD + AGP group. For TGF-*β*1 group, LX-2 cells were treated with TGF-*β*1 (10 ng/ml) for 24 hr. For PFD group, LX-2 cells were treated with TGF-*β*1 (10 ng/ml) for 24 hr and then were stimulated with PFD (1 mM) for 24 hr. For AGP group, LX-2 cells were treated with TGF-*β*1 (10 ng/ml) for 24 hr and then were stimulated with AGP (10 *μ*M) for 24 hr. For AGP + PFD group, LX-2 cells were treated with TGF-*β*1 (10 ng/ml) for 24 hr and then were stimulated with AGP (10 *μ*M) and PFD (1 mM) for 24 hr. Cells were subcultured once a day, and each experiment was carried out on logarithmic growth phase cells.

### 2.2. Chemicals and Reagents

PFD (purity >99%) was supplied by Medchem Express (MCE Co. Ltd., Shanghai, China). AGP (purity >99%) was obtained from Chengdu Herbpurify Co., Ltd. (Chengdu, China). Recombinant human TGF-*β*1 was furnished by Novoprotein (SinoBio, Shanghai, China). DPBS and Cell Proliferation Kit I (MTT) was provided by Beyotime Institute of Biotechnology (Haimen, China). Primary antibodies against *α*-SMA, collagen I, collagen IV, CTGF, p-Smad2, Smad2, p-Smad3, Smad3, TGF-*β*1, and *β*-actin were purchased from Wuhan Sanying Biotechnology (Wuhan, China), and all secondary antibodies were from Cell Signaling Technology (Cambridge, MA, USA). Dulbecco's Modified Eagle's Medium (DMEM) was from Gibco BRL (Carlsbad, CA, USA).

### 2.3. Animal Study

Male C57BL/6J mice were obtained from the Hunan Yuanhe Biotechnology Company Limited (Changsha, China). All reported experimental procedures followed the National Institutes of Health's guidelines for the care and use of experimental animals. This study was approved by the Experimental Animal Ethics Committee of the Hunan Yuanhe Biotechnology Co., Ltd., on March 7, 2023 (No. YH2023030701). The mice were randomly divided into five groups according to body weight (*n* = 6/group): The sham group received laparotomy without bile duct ligation (BDL); the model group received BDL operation by the common bile ducts which were ligated twice with 5.0 silk sutures and cut between the ligation; the model + PFD group received PFD gavage at a dose of 250 mg/kg/d after BDL; the model + AGP group treated AGP intraperitoneal injection at a dose of 5 mg/kg/d after BDL; and the model + PFD + AGP group received PFD gavage at a dose of 250 mg/kg/day and AGP intraperitoneal injection at a dose of 5 mg/kg/day after BDL. Eight-week-old mice subjected to the operation were anesthetized with tribromoethanol through intraperitoneal injection. The mice that received sham operation or BDL were euthanized via CO_2_ inhalation after 14 days. Blood samples were collected by cardiac puncture. Serum and liver tissues were collected for subsequent experiments.

### 2.4. MTT Assay

Cell viability was evaluated using an MTT assay. LX-2 cells (5 × 103 cells/well) in a logarithmic phase were inoculated into 96-well plates, and each group was provided with five auxiliary wells. After 24 hr of culture, the cells were attached to the wall. The next day, the original medium containing different drugs was changed, TGF-*β*1 (10 ng/ml), PFD (0–1.35 mM), and AGP (0–160 *μ*M), and the cells were incubated at 37°C for 24 hr in 5% CO_2_. Then, the cells were incubated with 50 *μ*L MTT solution for another 4 hr at 37°C. Finally, the absorbance at the wavelength of 570 nm was detected by MTT assay as the OD value in each group. The coefficient of drug interaction (CDI) was used to calculate synergy with the combination: CDI = AB/(A × B), where AB is the ratio of the combination group to the control group and A or B is the ratio of the single drug group to the control group. CDI < 1 indicates a synergistic effect, CDI = 1 indicates an additive effect, and CDI > 1 indicates an antagonistic effect.

### 2.5. Immunofluorescence Staining

Treated LX-2 cells were fixed with ice ethanol at −20°C for 20 min and blocked with 1% BSA for 1 hr at room temperature. Then, cells were incubated with anti-*α*-SMA (dilution 1 : 100), and anti-collagen I (dilution 1 : 100) at 4°C overnight. Cells were stained with DAPI to visualize the nuclei. Then, cells were incubated with the corresponding secondary antibodies (1 : 200, Wuhan Sanying Biotechnology, Wuhan, China) for 1 hr at room temperature. Finally, after washing, LX-2 cell nuclei (blue) were stained with DAPI and analyzed by a laser confocal microscope (magnification 600x, Olympus, FV10C-W3). According to the fluorescence intensity, the intensity of positive expression was observed.

### 2.6. Histopathological Analysis

Histopathological analysis fixed liver specimens in a 10% formalin solution and then dehydrated each tissue and embedded it in paraffin. Then, the liver tissues were cut into 4-*μ*m sections and stained with Masson's trichrome staining to evaluate the structural changes of the liver and the state of the fibrosis areas. These slices were observed under the microscope (magnification 600x, Olympus, FV 10 C-W 3), and the amount of collagen was quantified by ImageJ software.

### 2.7. ELISA Measurement

According to the manufacturer's instructions, the levels of inflammatory markers (IL-1 *β*, IL-6, and TNF-*α*) in treated LX-2 cells were measured by commercial ELISA kits. The concentrations of IL-1 *β*, IL-6, and TNF-*α* in the serum of mice were also detected by ELISA and standardized to protein concentration, as measured by the BCA test.

### 2.8. Real-Time QRT-PCR Assay

RNA was extracted from the treated LX-2 cells by TRIzol (Takara, Dalian, China) according to the manufacturer's protocol. Then, cDNA was acquired by SuperScript II reverse transcriptase (Invitrogen; Thermo Fisher Scientific, Inc.) of the extracted total RNA via the PrimeScript RT reagent Kit (Takara). Later, SYBR Green detection reagent (Promega Corporation, Madison, WI, USA) was applied for qRT-PCR analysis. Sequences of PCR primers were as follows: *α*-SMA, F: CGTTACTACTGCTGAGCGTG and R: TGAAGGATGGCTGGAACAGG; collagen I, F: GGACACAGAGGTTTCAGTGG and R: CAGTAGCACCATCATTTCCACG; Collagen IV, F: CTTCATTAGCAGGTGTGCGG and R: CGATGAATGGGGCGCTTCTA; CTGF, F: CTGGTCCAGACCACAGAGTG and R: TGCCCTTCTTAATGTTCTCTTCCA; IL-1*β*, F: TGCCACCTTTTGACAGTGATG and R: ATGTGCTGCTGCGAGATTTG; IL-6, F: ACTCACCTCTTCAGAACGAATTG and R: CCATCTTTGGAAGGTTCAGGTTG; TNF-*α*, F: TGTTCATCCG TTCTCTACCCA and R: CACTACTTCAGCGTCTCGT; and *β*-actin, F: TTCCTTCCTGGGCATGGAGTC and R: TTCCTTCCTGGGCATGGAGTC. Finally, the results were calculated as relative gene expression (fold change) using the 2^−(△△CT)^ method and normalized to *β*-actin RNA as internal controls.

### 2.9. Western Blot Analysis

Total proteins were extracted from treated LX-2 cells, and the protein content was measured using BCA Protein Assay (Thermo Scientific, Fremont, USA). The equivalent total proteins (25 *μ*g) were classified by SDS-PAGE and then transferred to PVDF (Millipore Corp., Bedford, MA, USA) membranes. The membranes were blocked with 5% nonfat milk in Tris-buffered saline (TBS) with Tween-20 at room temperature for 1 hr and subsequently incubated with primary antibodies at 4°C overnight. After washing three times × 10 min with 0.1% Tween–TBS, the membranes were incubated with secondary antibodies (1 : 5,000 dilutions of each antibody) at room temperature for 1 hr. *β*-Actin was selected as the internal control. An imaging system (Millipore, USA) was used to scan the obtained blots. The immunoblotted bands were visualized and quantified using image analysis software (Image Processing and Analysis in Java; NIH, Bethesda, MD, USA). The primary antibodies used were as follows: anti-*α*-SMA antibody (1 : 1,000 dilution), anti-collagen I antibody (1 : 1,000 dilution), anti-CTGF antibody (1 : 1,000 dilution), anti-p-Smad2 antibody (1 : 500 dilution), anti-Smad2 antibody (1 : 500 dilution), anti-p-Smad3 antibody (1 : 500 dilution), anti-Smad3 antibody (1 : 500 dilution), anti-TGF-*β*1 antibody (1 : 1,000 dilution), and anti-*β*-actin antibody (1 : 1,000 dilution).

### 2.10. Statistical Analyses

The results are presented as mean ± SEM. Unless otherwise specified, all experiments were repeated at least three times. All data were analyzed using SPSS Statistics 23.0, whereas GraphPad Prism 8.3.0 was used to generate diagrams and calculate the IC_50_ values. Data were analyzed by unpaired Student's *t*-test between the two groups and were analyzed by one-way analysis of variance (ANOVA) with Bonferroni's correction for multiple comparisons. The level of statistical significance was set at  ^*∗*^*P* < 0.05,  ^*∗∗*^*P* < 0.01, and  ^*∗∗∗*^*P* < 0.001.

## 3. Results

### 3.1. Effect of PFD, AGP, and PFD + AGP on the Viability of Lx-2 Cells

**Figure 1:**
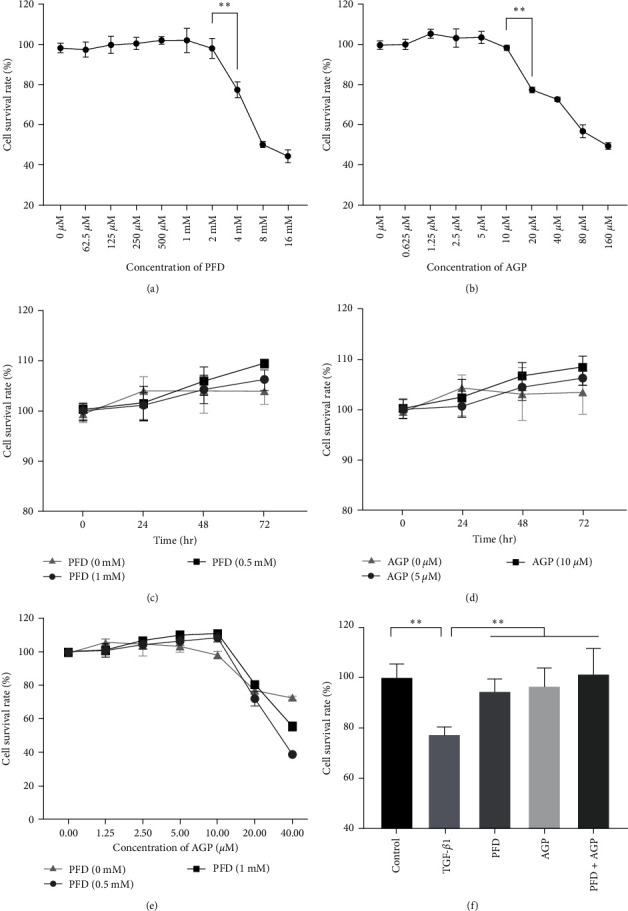
Effect of PFD, AGP, and PFD + AGP on the viability of Lx-2 cells. (a) Cell viability (%) of Lx-2 cells treated with PFD of various concentrations. (b) Cell viability (%) of Lx-2 cells treated with AGP of various concentrations. (c) Cell viability (%) of Lx-2 cells treated with PFD of various time gradients. (d) Cell viability (%) of Lx-2 cells treated with AGP of various time gradients. (e) Cell viability (%) of Lx-2 cells treated with PFD (0, 0.5, and 1 mM) and AGP of various concentrations. (f) LX-2 cells were treated with TGF-*β*1 (10 ng/ml) for 24 hr, then were stimulated with AGP (10 *μ*M) or/and PFD (1 mM) for 24 hr. Cell viability was measured by MTT assay.  ^*∗∗*^*P* < 0.01.

To determine the cytotoxicity of PFD, AGP, and PFD + AGP in Lx-2 cells, MTT assay was applied to measure the viability of Lx-2 cells exposed to PFD, AGP, and PFD + AGP at different doses and time gradients. As shown in Figure 1(a), PFD up to 2 mM did not affect cell viability, with the IC_50_ values of 10.74 mM. AGP up to 10 *μ*M had no obvious effect on cell death. However, obvious growth inhibition and toxicity of Lx-2 cells began to appear when the concentration of AGP reached more than 20 *μ*M, with the IC_50_ values of 125.0 *μ*M (Figure 1(b)). No matter 1 mM PFD or 10 *μ*M AGP had no obvious effect on cell viability within 72 hr (Figures 1(c) and 1(d)). Treatment with 1 mM PFD combined with 10 *µ*M AGP had no obvious effect on cell viability (Figure 1(e)). To determine whether cotreatment with PFD and AGP could produce synergistic effects on growth inhibition in LX-2 cells, the coefficient of drug interaction (CDI) was calculated. As shown in Table 1, 1 mM PFD and 10 *µ*M AGP yielded antagonistic interactions on growth inhibition in LX-2 cells (CDI > 1). Therefore, 1 mM PFD and 10 *µ*M AGP were used in the following experiments. In addition, the viability of Lx-2 cells induced by TGF-*β*1 decreased, whereas remarkable increases were observed in the viability of PFD, AGP, and PFD + AGP treated cells (Figure 1(f)).

### 3.2. Expression of *α*-SMA, Collagen I, and CTGF Was Significantly Upregulated in Activated LX-2 Cells

**Figure 2:**
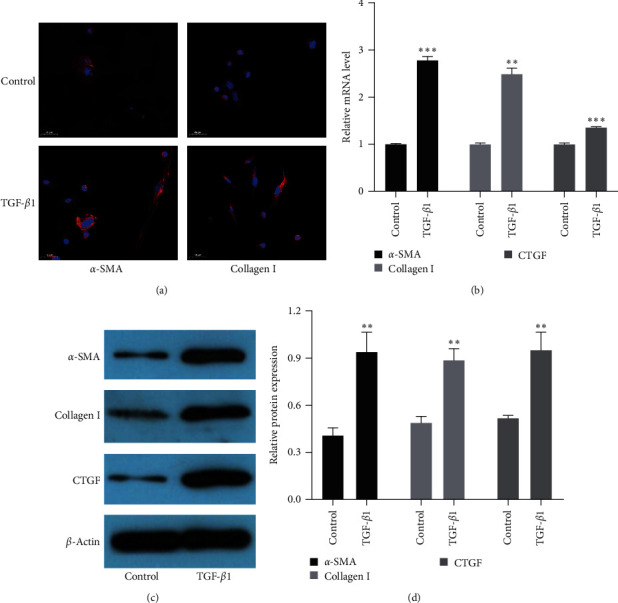
Expression of *α*-SMA, collagen I, and CTGF were significantly upregulated in activated LX-2 cells. (a) DAPI staining of *α*-SMA and collagen I (original magnification, ×200). (b) The mRNA levels of *α*-SMA, collagen I, and CTGF were detected by RT-qPCR. (c) The protein levels of *α*-SMA, collagen I, and CTGF were measured by Western blot. (d) The quantification of *α*-SMA, collagen I, and CTGF proteins expressions was measured by densitometry analysis.  ^*∗∗*^*P* < 0.01;  ^*∗∗∗*^*P* < 0.001.

To establish the cell model of liver fibrosis in BA, LX-2 cells were induced by TGF-*β*1 for 24 hr, and DAPI staining showed that *α*-SMA and collagen I accumulation was significantly increased in activated LX-2 cells (Figure 2(a)). Then, we used RT-qPCR to measure the expression of *α*-SMA, collagen I, and CTGF in cells. As shown in Figure 2(b), the expressions of *α*-SMA, collagen I, and CTGF dramatically increased in the TGF-*β*1 group compared to the control group. Furthermore, *α*-SMA, collagen I, and CTGF proteins were measured by Western blot. Interestingly, consistent with RT-qPCR results, the protein expressions of *α*-SMA, collagen I, and CTGF in LX-2 cells were significantly increased in the TGF-*β*1 group compared to the control group (Figures 2(c) and 2(d)). All these results indicated that the liver fibrosis model *in vitro* was successfully established.

### 3.3. Effect of PFD, AGP, and PFD + AGP on the Expressions of *α*-SMA, Collagen I, and CTGF in Activated LX-2 Cells

**Figure 3:**
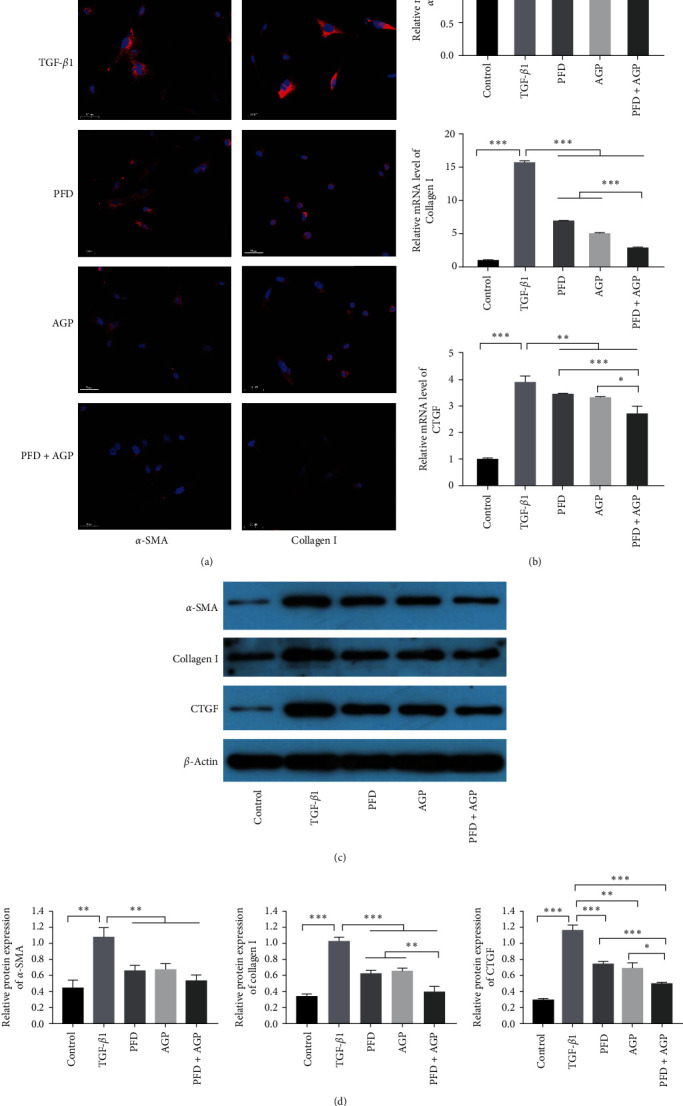
PFD, AGP, and PFD + AGP inhibited the expressions of *α*-SMA, collagen I, and CTGF in activated LX-2 cells. (a) DAPI staining of *α*-SMA and collagen I (original magnification, ×200). (b) The mRNA levels of *α*-SMA, collagen I, and CTGF were detected by RT-qPCR. (c) The protein levels of *α*-SMA, collagen I, and CTGF were measured by Western blot. (d) The quantification of *α*-SMA, collagen I, and CTGF proteins expressions was measured by densitometry analysis.  ^*∗*^*P* < 0.05;  ^*∗∗*^*P* < 0.01;  ^*∗∗∗*^*P* < 0.001.

To investigate the effects of PFD, AGP, and PFD + AGP on liver fibrosis, we used TGF-*β*1 to build the cell model of activated LX-2. DAPI staining showed PFD, AGP, and PFD + AGP treatment dramatically decreased *α*-SMA and collagen I deposition compared to the TGF-*β*1 group (Figure 3(a)). Then, we used RT-qPCR to measure the expression of fibrotic markers (*α*-SMA, collagen I, and CTGF) in activated LX-2 cells. As shown in Figure 3(b), the expression levels of *α*-SMA, collagen I, and CTGF dramatically decreased in the PFD, AGP, and PFD + AGP groups compared to the TGF-*β*1 group. Interestingly, treatment with PFD combined with AGP obviously attenuated the expressions of collagen I and CTGF, as compared to the PFD or AGP monotherapy group. Treatment with PFD combined with AGP obviously attenuated the expressions of *α*-SMA, as compared to the PFD monotherapy group. However, treatment with PFD combined with AGP did not obviously attenuate the expressions of *α*-SMA, as compared to the AGP monotherapy group. Furthermore, the protein levels of *α*-SMA, collagen I, and CTGF were measured by Western blot. Interestingly, consistent with RT-qPCR results, the protein expressions of collagen I, *α*-SMA, and CTGF in activated LX-2 cells were significantly decreased in the PFD, AGP, and PFD + AGP groups compared to the TGF-*β*1 group. In addition, treatment with PFD combined with AGP obviously attenuated the protein expressions of collagen I and CTGF, as compared to the PFD or AGP monotherapy group. However, compared with the PFD or AGP monotherapy, the synergistic treatment of PFD and AGP did not increase the inhibition of *α*-SMA in activated LX-2 cells (Figures 3(c) and 3(d)). Overall, these results revealed that PFD, AGP, and PFD + AGP inhibited the expressions of CTGF, collagen I, and *α*-SMA in activated LX-2 cells. The combination of PFD and AGP exerted superior inhibition effects on hepatic stellate cell activation, as compared to monotherapy.

### 3.4. Effects of PFD, AGP, and PFD + AGP on Inflammatory Biomarkers in Activated LX-2 Cells

**Figure 4:**
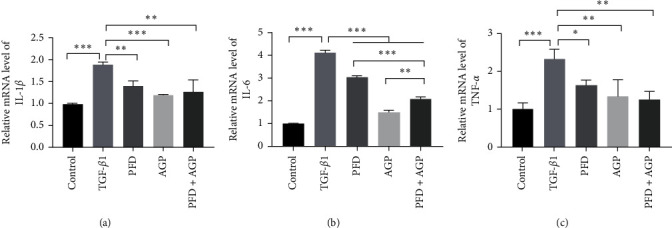
PFD, AGP, and PFD + AGP inhibited the expressions of inflammatory biomarkers in activated LX-2 cells were shown by mRNA expressions of (a) IL-1*β*/*β*-actin, (b) IL-6/*β*-actin, and (c) TNF-*α*/*β*-actin.  ^*∗*^*P* < 0.05;  ^*∗∗*^*P* < 0.01;  ^*∗∗∗*^*P* < 0.001.

The expression levels of IL-1*β*, IL-6, and TNF-*α* in activated LX-2 cells were measured by RT-qPCR. As presented in Figures 4(a), 4(b), and 4(c), the expression levels of IL-1*β*, IL-6, and TNF-*α* were significantly increased in the TGF-*β*1 group, respectively, as compared to the control group. The expression levels of IL-1*β*, IL-6, and TNF-*α* dramatically decreased in the PFD, AGP, and PFD + AGP group compared to the TGF-*β*1 group. Interestingly, treatment with PFD combined with AGP obviously attenuated the expressions of IL-6, as compared to the PFD monotherapy group. However, treatment with PFD combined with AGP did not obviously attenuate the expressions of IL-1*β* and TNF-*α*, as compared to the PFD or AGP monotherapy group.

### 3.5. Effect of PFD, AGP, and PFD + AGP on the Phosphorylation of Smad2 and Smad3 in Activated LX-2 cells in the TGF-*β*/Smad Signaling Pathway

**Figure 5:**
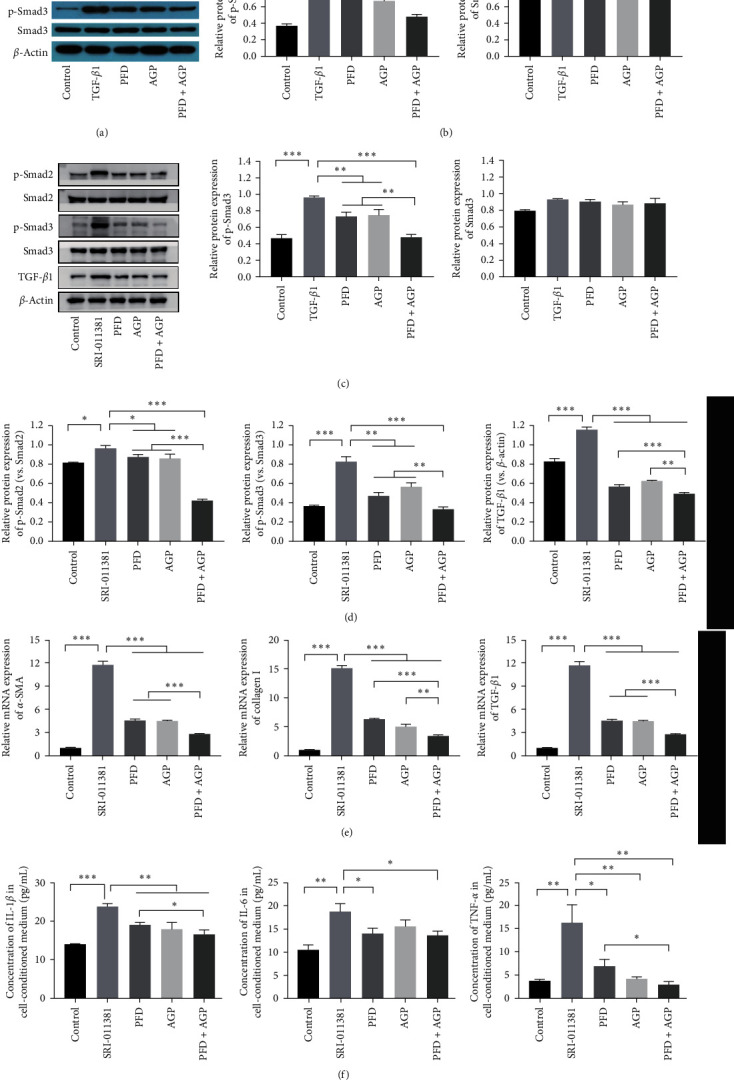
PFD, AGP, and PFD + AGP attenuated the phosphorylation of Smad2 and Smad3 in activated LX-2 cells in the TGF-*β*/Smad signaling pathway. (a) and (c) The protein expression of p-Smad2, Smad2, p-Smad3, Smad3, and TGF-*β*1 were detected by Western blot. (b) The quantification of p-Smad2, Smad2, p-Smad3, and Smad3 protein expressions were measured by densitometry analysis. (d) The ratio of p-Smad2/Smad2, p-Smad3/Smad3, and TGF-*β*1/*β*-actin were measured by densitometry analysis. (e) The mRNA levels of *α*-SMA, collagen I, and TGF-*β*1 were detected by RT-qPCR. (f) The levels of IL-1 *β*, IL-6, and TNF-*α* in treated LX-2 cells were measured by ELISA.  ^*∗*^*P* < 0.05;  ^*∗∗*^*P* < 0.01;  ^*∗∗∗*^P < 0.001.

To verify whether the TGF-*β*/Smad signaling pathway was related to PFD or/and AGP ameliorates hepatic stellate cell activation. The efficacy of PFD, AGP, and PFD + AGP on the TGF-*β*/Smad signal pathway was evaluated in activated LX-2 cells. As represented in Figures 5(a) and 5(b), the expressions of p-Smad2 and p-Smad3 distinctly decreased in activated LX-2 cells after treatment with PFD, AGP, and PFD + AGP in comparison with the TGF-*β*1 group. However, the total protein expressions of Smad2 and Smad3 had no obvious change before and after PFD, AGP, and PFD + AGP treatment. Strikingly, treatment with PFD combined with AGP obviously attenuated the expressions of p-Smad2 and p-Smad3, as compared to the PFD or AGP monotherapy group. To future verify whether PFD or/and AGP are involved in TGF-*β*/Smad signaling, we used the TGF-*β*/Smad signaling agonist SRI-011381 to treat LX-2 cells for 24 hr in the SRI-011381 group. LX-2 cells were treated with SRI-011381 (10 *μ*M) + PFD (1 mM), SRI-011381 (10 *μ*M) + AGP (10 *μ*M), and SRI-011381 (10 *μ*M) + PFD (1 mM) + AGP (10 *μ*M) for 24 hr in the PFD group, AGP group, and AGP + PFD group, respectively. As shown in Figures 5(c) and 5(d), SRI-011381 significantly increased the expression of TGF-*β*1, p-Smad2, and p-Smad3 in LX-2 cells compared to the control group. On the contrary, PFD or/and AGP obviously reversed these changes induced by SRI-011381. Interestingly, treatment with PFD combined with AGP obviously attenuated the expressions of p-Smad2, p-Smad3, and TGF-*β*1, as compared to the PFD or AGP monotherapy group. These data showed that PFD or/and AGP attenuated HSC activation by inhibiting the TGF-*β*/Smad signaling pathway. As shown in Figures 5(e) and 5(f), SRI-011381 significantly induced the hepatic fibrosis markers (*α*-SMA, collagen I, and TGF-*β*1) and inflammatory markers (IL-1*β*, IL-6, and TNF-*α*), compared to the control group. On the contrary, PFD or/and AGP markedly reversed these changes induced by SRI-011381. Moreover, compared with monotherapy, combination therapy with PFD and AGP synergistically inhibited the expression of the hepatic fibrosis markers (*α*-SMA, collagen I, and TGF-*β*1) and inflammatory markers (IL-1*β*, IL-6, and TNF-*α*) in activated LX-2 cells.

### 3.6. Effects of PFD, AGP, and PFD + AGP on Liver Fibrosis and Inflammation in the Mice Model of BA.

**Figure 6:**
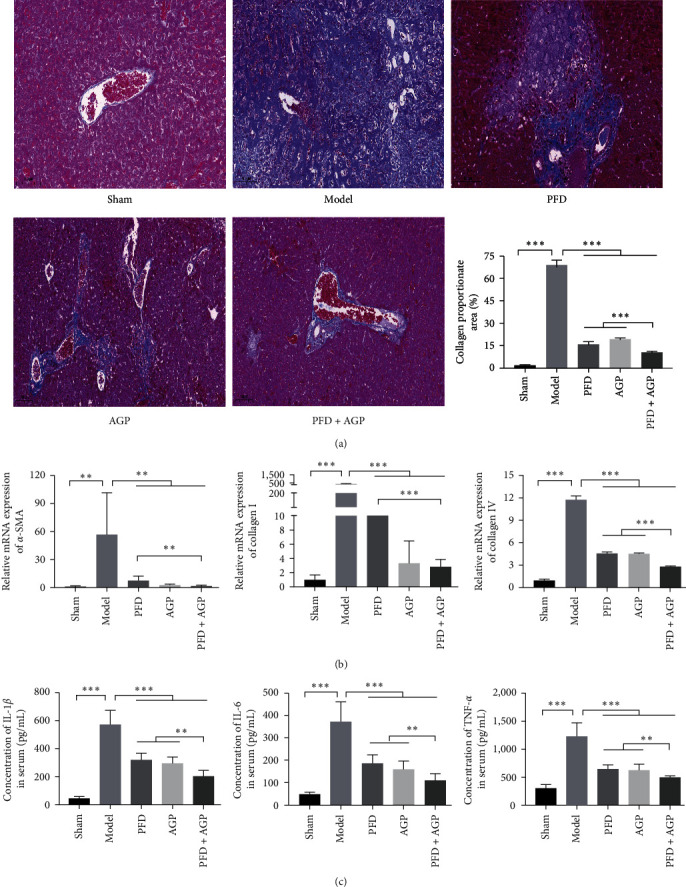
PFD, AGP, and PFD + AGP inhibited liver fibrosis and inflammation in the mice model of BA. (a) Masson's trichrome staining (bar = 100 *μ*m) of collagen in liver tissue in different groups. (b) The mRNA levels of *α*-SMA, collagen I, and collagen IV were detected by RT-qPCR. (c) The concentrations of IL-1 *β*, IL-6, and TNF-*α* in the serum of mice were measured by ELISA.  ^*∗∗*^*P* < 0.01;  ^*∗∗∗*^*P* < 0.001.

To investigate the effects of PFD, AGP, and PFD + AGP on liver fibrosis, we used the method of BDL to build the mice model of BA *in vivo*. As shown in Figure 6(a), Masson's trichrome staining showed that the distribution of hepatocytes in the sham-operated group was normal, the nuclei of hepatocytes were clearly visible, and there were no obvious collagen fibers. However, many liver cells were destroyed, and a large number of collagen fibers appeared in the model group. Interestingly, in the PFD, AGP, and PFD + AGP groups, the extent of the collagen fibers in hepatic tissues was apparently reduced compared to the model group. Strikingly, treatment with PFD combined with AGP obviously attenuated the accumulation of collagen fibers, as compared to the PFD or AGP monotherapy group. Then, we used RT-qPCR to measure the expression of fibrotic markers (*α*-SMA, collagen I, and collagen IV) in liver tissue. As shown in Figure 6(b), the expression levels of *α*-SMA, collagen I, and collagen IV dramatically decreased in the PFD, AGP, and PFD + AGP groups compared to the model group. Interestingly, treatment with PFD combined with AGP obviously attenuated the expressions of *α*-SMA, collagen I, and collagen IV, as compared to the PFD or AGP monotherapy group. Furthermore, the levels of IL-1 *β*, IL-6, and TNF-*α* in the serum of BA mice were measured by ELISA (Figure 6(c)). The concentrations of expressions of IL-1 *β*, IL-6, and TNF-*α* in the serum of mice were significantly decreased in the PFD, AGP, and PFD + AGP groups compared to the TGF-*β*1 group. In addition, treatment with PFD combined with AGP obviously attenuated the levels of IL-1 *β*, IL-6, and TNF-*α*, as compared to the PFD or AGP monotherapy group. Altogether, these results revealed that PFD, AGP, and PFD + AGP inhibited liver fibrosis and inflammation in the mice model of BA. The combination of PFD and AGP exerted superior antifibrosis and anti-inflammatory effects, as compared to monotherapy.

## 4. Discussion

The exact pathogenesis of BA remains largely unclear. Persistent inflammation, matrix deposition, immune response, and decompensated angiogenesis all contribute to liver fibrosis progression [15]. Although the long-term outcome of BA patients is gradually improving, treatment of liver fibrosis remains a major clinical challenge. At present, the antifibrosis drug effect of BA is not satisfactory. Therefore, a novel and effective strategy for the treatment of liver fibrosis is urgently needed. Combination therapies are becoming more popular because they can improve clinical effects by targeting a variety of ways and have potential synergy. However, the antihepatic fibrosis effects of PFD and AGP are rarely reported. As far as we know, our findings for the first time suggest that the beneficial potential of PFD combined with AGP in regression of HSC activation induced by TGF-*β*1 and liver fibrosis in the mice model of BA, with its regulatory effects on TGF-*β*/Smad signaling pathways.

HSCs are the key effector of hepatic fibrosis and the main source of ECM. Inhibition of activated HSC is considered a new treatment strategy for hepatic fibrosis [16]. The present study indicated that the expression of CTGF, collagen I, collagen IV, and *α*-SMA was significantly increased in activated Lx-2 cells. *α*-SMA is considered a marker of HSC activation, and the level of a-SMA in patients with BA is positively correlated with the severity of liver fibrosis [17]. Collagen I is the main component of ECM, mainly synthesized and secreted by fibroblasts, and its excessive accumulation is the pathological change of liver fibrosis. A higher perisinus collagen I deposition in initial biopsies is associated with a poor prognosis of BA after surgical treatment [18]. CTGF mainly affects connective tissue cells, and it is lowly expressed in stromal cells under normal conditions. CTGF is an early marker of collagen synthesis in the fibrotic process, and blocking it can reverse fibrosis [19]. With the aggravation of liver fibrosis in BA patients, the expression level of CTGF in liver tissue gradually increased, suggesting that CTGF is closely related to liver fibrosis [5]. Our data showed that combination therapy with PFD and AGP, PFD, or AGP monotherapy suppressed the expressions of CTGF, collagen I, and *α*-SMA in activated LX-2 cells, and the expressions of *α*-SMA, collagen I, and collagen IV in mice model of BA. These results indicate that PFD and AGP alone or in combination can directly act on activated HSC and alleviate hepatic fibrosis *in vivo*. Compared with the AGP or PFD monotherapy, combination therapy with PFD and AGP resulted in a significant decrease in the expression of collagen I, collagen IV, and CTGF, which indicated that combined treatment was superior to monotherapy in alleviating liver fibrosis.

BA involves three major fields of disease: inflammation, cholestasis, and liver fibrosis. Studies have shown that many inflammatory mediators are released after the activation of various inflammatory pathways in children with BA. Inflammatory mediators cause damage to liver cells and intrahepatic bile duct epithelial cells and promote the release of inflammatory mediators, which promote the transformation of HSCs into myofibroblasts and lead to the formation of liver fibrosis [20]. Therefore, inflammatory cytokines play a vital role in the process of hepatic fibrosis of biliary atresia. In BA patients and BA model rats, IL-6, IL-1*β*, and TNF-*α* significantly increased [21, 22, 23]. In the process of liver injury, IL-6 is released from bile duct epithelial cells, which directly promotes cell regeneration and proliferation [24]. IL-6 was confirmed as a biomarker of the severity of liver cirrhosis in BA [25]. *In vivo* data coupled with *in vitro* data suggested that the proinflammatory state, particularly through TNF-*α* and IL-1*β*, drives dysregulation of bile acid homeostasis and transport [19]. In addition to the antifibrosis effect, PFD also plays an anti-inflammatory role by inhibiting the synthesis of inflammatory cytokines such as IL-1*β*, IL-6, TNF-*α*, and IFN-*γ* [20]. AGP is known as a natural antibiotic, and its anti-inflammatory effect has long been concerned. Recent research has shown that AGP can suppress inflammatory mediator production of IL-1*β*, IL-6, TNF-*α*, and IL-1*α* in the treatment of gouty inflammation [26]. In the present study, PFD and AGP treatment separately and together suppressed the expressions of IL-6, IL-1*β*, and TNF-*α* in activated Lx-2 cells and mice model of BA and showed good anti-inflammatory effects. Compared with PFD or AGP monotherapy, combination treatment of PFD and AGP showed a stronger anti-inflammatory effect in this experiment.

TGF-*β*1 is the most widely studied subtype of liver fibrosis which is produced by intrahepatic bile duct epithelial cells. TGF-*β*1 released by necrotic hepatocytes during liver injury may be the first signal of activating adjacent stationary HSC, leading to its transdifferentiation into myofibroblasts. TGF-*β*1 is considered an effective cytokine to promote fibrosis and is closely related to liver fibrosis of BA [27]. TGF-*β*/Smad signaling pathway plays an important role in the occurrence and development of liver fibrosis, in which TGF-*β* interacts with T*β*RI, mediating to phosphorylation of downstream proteins (Smad2 and Smad3) forming a complex with Smad4 (the common mediator SMAD protein), transferring to the nucleus and regulating the expression of target genes, including *α*-SMA and collagen I [27]. Smad7, as an antagonist protein of Smad, can bind to T*β*RI to interfere with the phosphorylation of Smad2/Smad3, forming a negative feedback loop of TGF-*β*1/Smad pathway to inhibit the development of fibrosis [28]. Our research shows that PFD or/and AGP can inhibit the TGF-*β*/Smad signaling pathway by inhibiting p-Smad2 and p-Smad3. Notably, compared with the PFD or AGP monotherapy, PFD combined with AGP can significantly inhibit the phosphorylation of Smad2 and Smad3 in HSCs. Studies have confirmed that blocking the Smad2/3 signaling pathway in rodents can prevent liver fibrosis, suggesting that blocking this signaling pathway is a possible method to treat liver fibrosis in BA [29]. Both PFD and AGP can also inhibit TGF-*β*/Smad signaling pathway by increasing the expression of smad7 [30, 31]. TGF-*β*/Smad signaling pathway may be an attractive target for the prevention and treatment of liver fibrosis. Our research shows that the combination therapy consisting of PFD and AGP plays a synergistic role in improving liver fibrosis, and this may provide a new and effective treatment strategy for liver fibrosis of BA.

## 5. Conclusion

**Figure 7:**
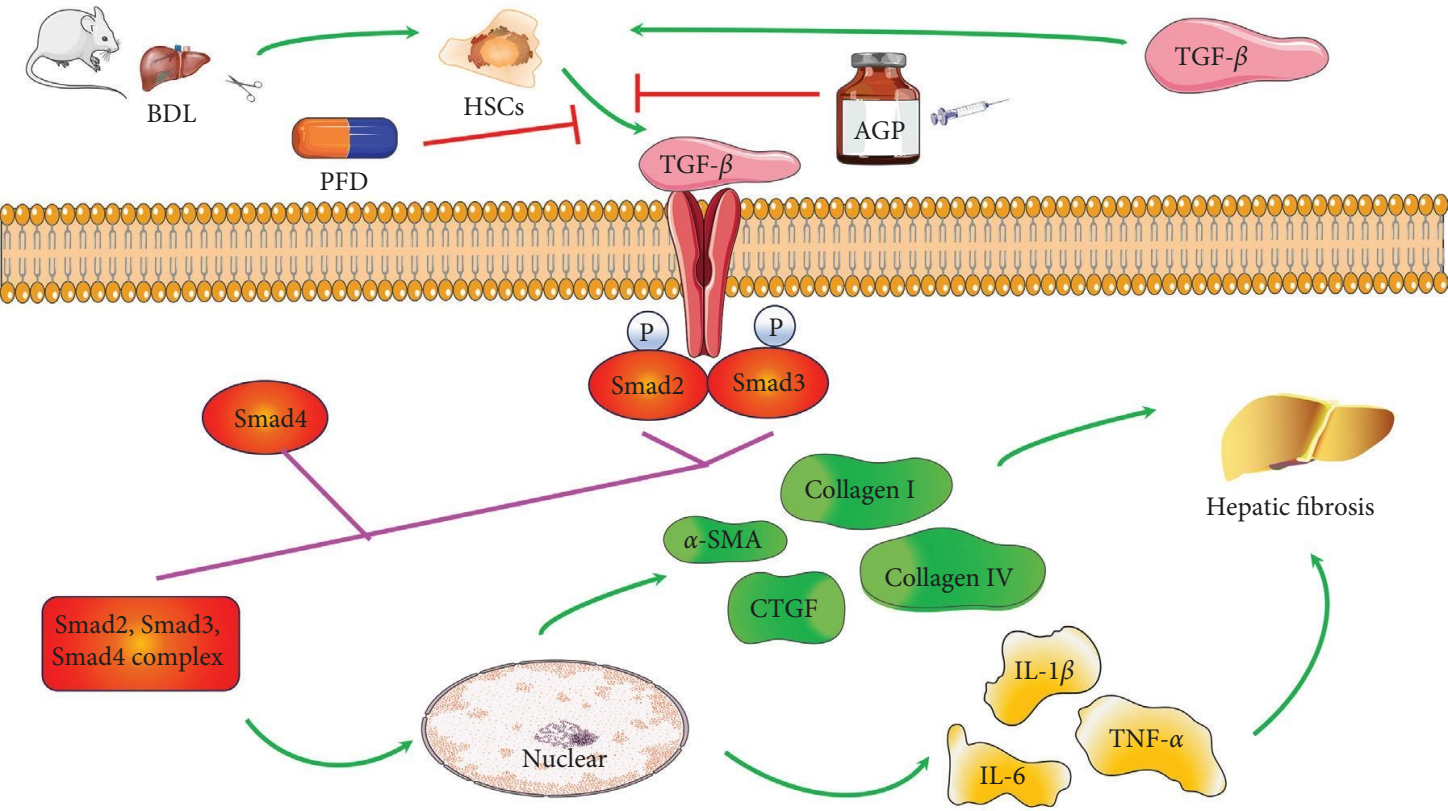
The proposed mechanism of PFD and/or AGP ameliorates hepatic stellate cell activation and liver fibrosis by mediating the TGF-*β*/Smad signaling pathway.

Our research results showed that both PFD and AGP can exert good antifibrosis and anti-inflammatory effects. Compared with monotherapy, PFD combined with AGP exerted superior antifibrosis and anti-inflammatory effects. In addition, PFD or/and AGP inhibited the activation of HSCs by blocking the TGF-*β*/Smad signaling pathway, and the combined treatment of PFD and AGP synergistically inhibited the phosphorylation of Smad2 and Smad3 (Figure 7). Therefore, a combination of PFD and AGP may be a potential treatment strategy for liver fibrosis in BA.

## Figures and Tables

**Table 1:** Dosage inhibitory effects of PFD and AGP on LX-2 cells (*n* = 4).

Drug	Concentration	Growth inhibitory effects (OD)	CDI
PFD	0 mM	0.995 ± 0.024	—
	0.5 mM	1.001 ± 0.022	—
	1 mM	1.004 ± 0.004	—
PFD + AGP	0 mM + 10 *µ*M	0.980 ± 0.012	—
	0.5 mM + 10 *µ*M	1.086 ± 0.034	1.108 ± 0.065
	1 mM + 10 *µ*M	1.111 ± 0.015	1.129 ± 0.025

CDI value <1, = 1, or >1 indicates that the drugs are synergistic, additive, or antagonistic, respectively.

## Data Availability

The data used to support the findings of this study are included within the article.

## Abbreviations

BA: Biliary atresia
HSCs: Hepatic stellate cells
PFD: Pirfenidone
AGP: Andrographolide
TGF-*β*1: Transforming growth factor-*β*1
ECM: Extracellular matrix
CTGF: Connective tissue growth factor
BDL: Bile duct ligation.
